# 

**Published:** 1891

**Authors:** 


### INTERMITTENT FEVER.

BY

P. P. WELLS, M. D.

TOGETHER WITH THE

REPERTORY OF CARL VON BŒNNINGHAUSEN, M. D.,

Also Translated by P. P. WELLS, M. D.

---

ISSUED AS A SUPPLEMENT TO

THE HOMŒOPATHIC PHYSICIAN.

---

PHILADELPHIA:
PUBLISHED BY
THE HOMŒOPATHIC PHYSICIAN,
1125 SPRUCE STREET,
1891.

